# 212. Effectiveness of Empirical Piperacillin/tazobactam Compared to Cefepime for Bloodstream Infection Caused by Gram-Negative Bacilli

**DOI:** 10.1093/ofid/ofad500.285

**Published:** 2023-11-27

**Authors:** Young Ho Lee, Jinyoung Yang, Jae-Hoon Ko, Sun Young Cho, Heejae Huh, Nam Yong Lee, Cheol-In Kang, Doo Ryeon Chung, Kyong Ran Peck, Jae-Hoon Song, Kyungmin Huh

**Affiliations:** Samsung Medical Center, Seoul, Seoul-t'ukpyolsi, Republic of Korea; Samsung Medical Center, Seoul, Seoul-t'ukpyolsi, Republic of Korea; Samsung Medical Center, Seoul, Seoul-t'ukpyolsi, Republic of Korea; Samsung Medical Center, Seoul, Korea, Seoul, Seoul-t'ukpyolsi, Republic of Korea; Department of Laboratory Medicine and Genetics, Samsung Medical Center, Sungkyunkwan University School of Medicine, Seoul, Seoul-t'ukpyolsi, Republic of Korea; Samsung Medical Center, Seoul, Seoul-t'ukpyolsi, Republic of Korea; Samsung Medical Center, Seoul, Seoul-t'ukpyolsi, Republic of Korea; samsung medical center, Seoul, Seoul-t'ukpyolsi, Republic of Korea; Samsung Medical Center, Seoul, Seoul-t'ukpyolsi, Republic of Korea; Asia Pacific Foundation for Infectious Diseases, Seoul, Seoul-t'ukpyolsi, Republic of Korea; samsung medical center, Seoul, Seoul-t'ukpyolsi, Republic of Korea

## Abstract

**Background:**

Piperacillin/tazobactam (PTZ) and cefepime (CFP) are commonly used antibiotics for healthcare-associated infections and immunocompromised patients. However, few studies have compared the clinical effectiveness of these two antibiotics. Thus, we compared the effectiveness of empirical PTZ and CFP in patients with monomicrobial blood-stream infection (BSI) caused by gram-negative bacilli.

**Methods:**

We conducted a retrospective cohort study of consecutive, non-duplicate monomicrobial BSIs caused by 1 of 4 taxa (*Escherichia coli, Klebsiella pneumonia, Pseudomonas aeruginosa* and *Acinetobacter* species) identified from a nationwide, prospective surveillance (Korean Antimicrobial Resistance Surveillance Network [KARS-Net]). Patients who were treated with PTZ or CFP as the only active empirical antibiotic were included. The primary outcome was all-cause, in-hospital 30-day mortality. Logistic regression with backward selection was used to adjust for imbalance in baseline characteristics.

**Results:**

A total of 120 cases were included, among which 87 (72.5%) were treated with PTZ and 33 (27.5%) with CFP. Underlying renal disease was more common in the PTZ group whereas malignancy, septic shock, and neutropenia were more common in the CFP group. (Table 1). All-cause 30-day in-hospital mortality was comparable between the two groups (16.1% vs 12.1%, p=0.777; table 2), as well as attributable mortality. However, the early clinical response at 72 hr was significantly more common in the patients treated with CFP (25.3% vs 57.6%, p=0.001)

The choice of empirical antibiotics was not significantly associated with mortality in a multivariable logistic regression model (Table 3). Older age (≥65 years; adjusted OR. 16.24; 95% CI, 2.21-119.60), lower ECOG (aOR, 2.91 per 1 point, 95% CI, 1.35-6.25), high PITT score (aOR, 1.68; 95% CI, 1.17-6.25) and chronic liver disease (aOR, 39.70; 95% CI, 2.56-615.13) were significant independent risk factors for 30-day in-hospital mortality.

**Figure:**
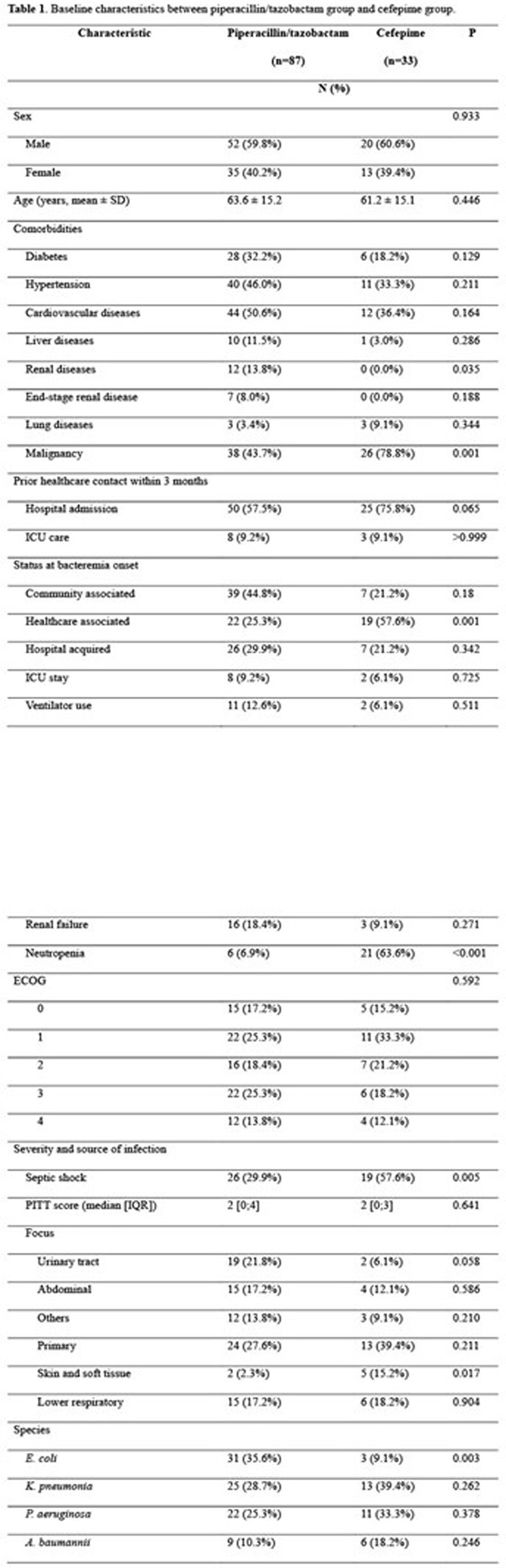

**Figure:**
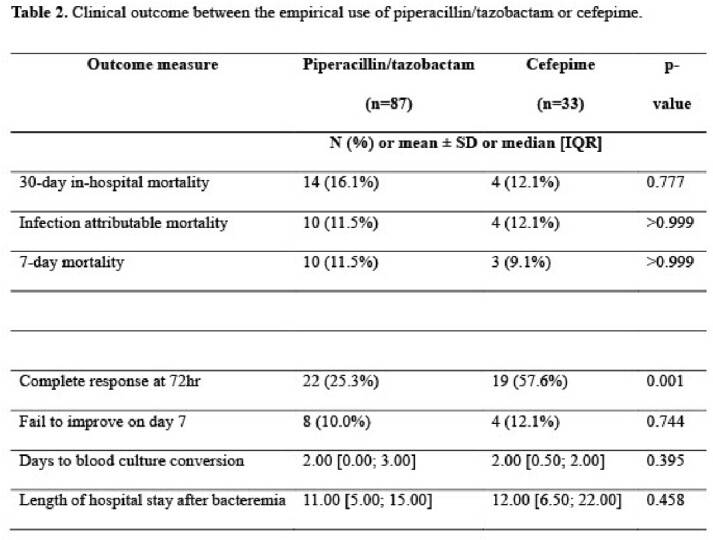

**Figure:**
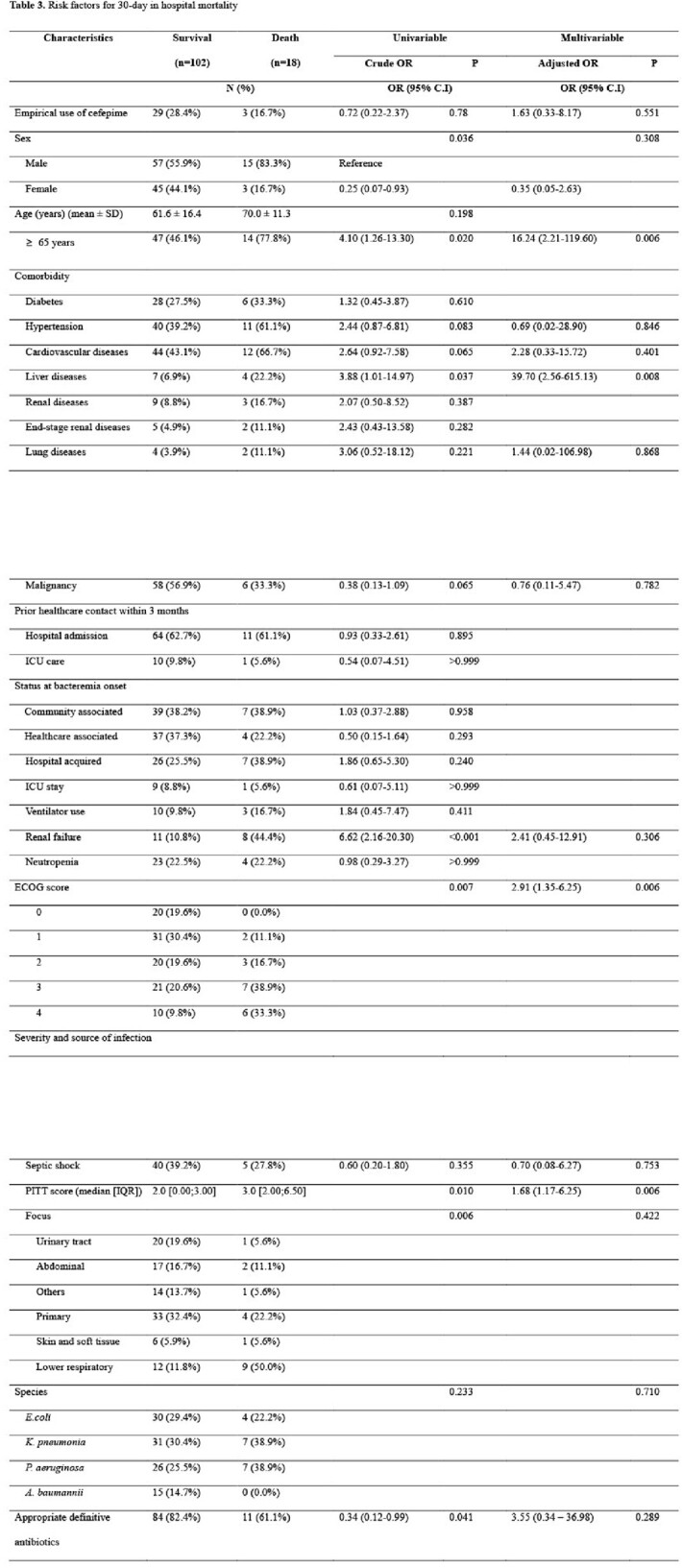

**Conclusion:**

In patients with BSI caused by gram-negative bacilli, empirical use of either PTZ or CFP was associated with comparable clinical outcomes.

**Disclosures:**

**All Authors**: No reported disclosures

